# GSK3A Is Redundant with GSK3B in Modulating Drug Resistance and Chemotherapy-Induced Necroptosis

**DOI:** 10.1371/journal.pone.0100947

**Published:** 2014-07-01

**Authors:** Emanuela Grassilli, Leonarda Ianzano, Sara Bonomo, Carola Missaglia, Maria Grazia Cerrito, Roberto Giovannoni, Laura Masiero, Marialuisa Lavitrano

**Affiliations:** 1 Department of Surgery and Traslational Medicine, Medical School, University of Milano-Bicocca, via Cadore 48, Monza, Italy; 2 BiOnSil srl, via Cadore 48, Monza, Italy; Federico II University of Naples, Italy, Italy

## Abstract

Glycogen Synthase Kinase-3 alpha (GSK3A) and beta (GSK3B) isoforms are encoded by distinct genes, are 98% identical within their kinase domain and perform similar functions in several settings; however, they are not completely redundant and, depending on the cell type and differentiative status, they also play unique roles. We recently identified a role for GSK3B in drug resistance by demonstrating that its inhibition enables necroptosis in response to chemotherapy in p53-null drug-resistant colon carcinoma cells. We report here that, similarly to GSK3B, also GSK3A silencing/inhibition does not affect cell proliferation or cell cycle but only abolishes growth after treatment with DNA-damaging chemotherapy. In particular, blocking GSK3A impairs DNA repair upon exposure to DNA-damaging drugs. As a consequence, p53-null cells overcome their inability to undergo apoptosis and mount a necroptotic response, characterized by absence of caspase activation and RIP1-independent, PARP-dependent AIF nuclear re-localization. We therefore conclude that GSK3A is redundant with GSK3B in regulating drug-resistance and chemotherapy-induced necroptosis and suggest that inhibition of only one isoform, or rather partial inhibition of overall cellular GSK3 activity, is enough to re-sensitize drug-resistant cells to chemotherapy.

## Introduction

Two different GSK3 isoforms, GSK3A and GSK3B, encoded by distinct genes, but 98% identical within their kinase domain, are expressed in mammalian cells [1]. Both isoforms perform similar functions in several settings, but they are not completely redundant as demonstrated by gene knockout studies. In fact, GSK3A is unable to rescue the lethal phenotype of GSK3B null mice: the animals die during embryogenesis as a result of liver degeneration caused by widespread hepatocyte apoptosis, where excessive TNF-alpha-mediated cell death occurs, due to reduced NFkB function [2]. On the other hand, GSK3A null mice are viable and show metabolic defects – such as enhanced glucose and insulin sensitivity and reduced fat mass - which cannot be counteracted by the beta isofom [3]. Moreover, GSK3A KO mice undergo premature death showing acceleration of age-related pathologies, accompanied by marked activation of mTORC1 and associated suppression of autophagy markers, indicating that the alpha isoform is a critical regulator of mTORC1, autophagy, and aging [4].

So far distinct roles for GSK3A and GSK3B have been identified in developmental and differentiation processes [5], as well as in regulation of transcriptional activation [6]. Functional redundancy instead has been demonstrated in the control of several regulatory proteins, in the production of beta-amyloid peptides associated with Alzheimer's disease and in cell cycle and proliferation. In the latter, both isoforms play an anti-proliferative role by promoting APC-dependent phosphorylation of β-catenin - a transcription factor positively regulating Myc and cyclin D1 expression – therefore targeting it to proteasome-mediated degradation [7]. Either redundant or distinct functions of the two isoforms have been demonstrated in cell survival, depending on the cell type [2], [8], [9]. In particular, a lot of data are being accumulated about the beta isoform acting as a tumor suppressor in some cancers while potentiating tumoral growth in others: for example, GSK3B activation can be crucial in mediating caspase-dependent apoptosis by contributing to p53 activation in certain epithelial cancers [10], whereas its inhibition arrests pancreatic tumor growth in vivo [11] and is synthetically lethal with MLL oncogene defects in a subset of human leukemia [12]. Moreover, in the experimental systems where GSK3B plays an oncogenic role its targeting has been proved useful, either alone on in combination with chemotherapy, to induce or increase tumor cells death [13], [14]. However, very few reports addressed the role of the alpha isoform in cancer cells growth/survival: so far, NFkB-dependent pro-survival effect has been demonstrated to be mediated either by GSK3A or GSK3B in pancreatic cancer cells [9] whereas GSK3A, but not GSK3B, has been identified as a therapeutic target in melanoma [15]. Therefore, very little is known about GSK3A role in cancer cells.

We recently identified a role for GSK3B in drug resistance by finding that its inhibition in p53-null, drug-resistant colon carcinoma cells re-sensitize them to chemotherapy by unleashing RIP1-independent necroptosis in response to DNA damaging agents [16]. Here we report that GSK3A is functionally redundant with GSK3B in modulating drug resistance and chemotherapy-induced necroptosis.

## Results

### GSK3A silencing in p53-null colon carcinoma cell lines does not affect proliferation but modifies the response to DNA-damaging chemotherapy

**Figure 1:**
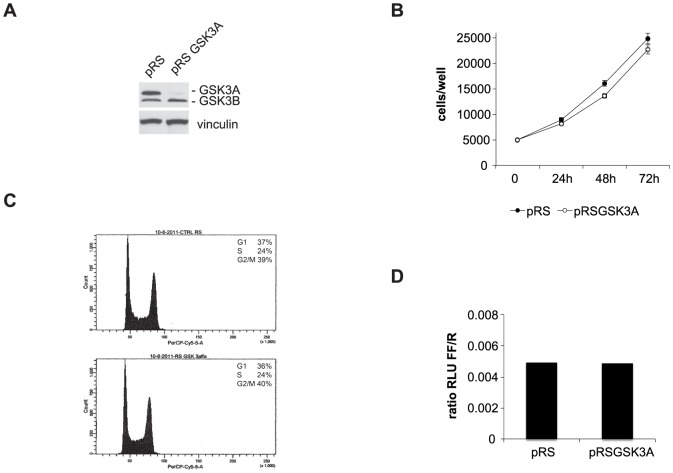
GSK3A silencing in p53-null colon carcinoma cell lines does not affect proliferation or cell cycle. **A**) Western blot on total lysates (30 µg) from HCT116p53KO cells stably infected with retroviral empty (pRS) and GSK3A shRNA-expressing (pRSGSK3A) vectors. **B**) growth curve of HCT116p53KO-pRS and -pRSGSK3A cells. **C**) DNA content and percentage of cells in G1, S phase and G2/M of HCT116p53KO-pRS and -pRSGSK3Aas evaluated after PI staning and flow cytometric analysis. **D**) β-catenin activity as evaluated by reporter assay 48 hrs after co-transfection with a luciferase-encoding vector driven by a β-catenin-dependent promoter. RLU  =  Relative Light Units. encoding vector (pRSGSK3B).

**Figure 2:**
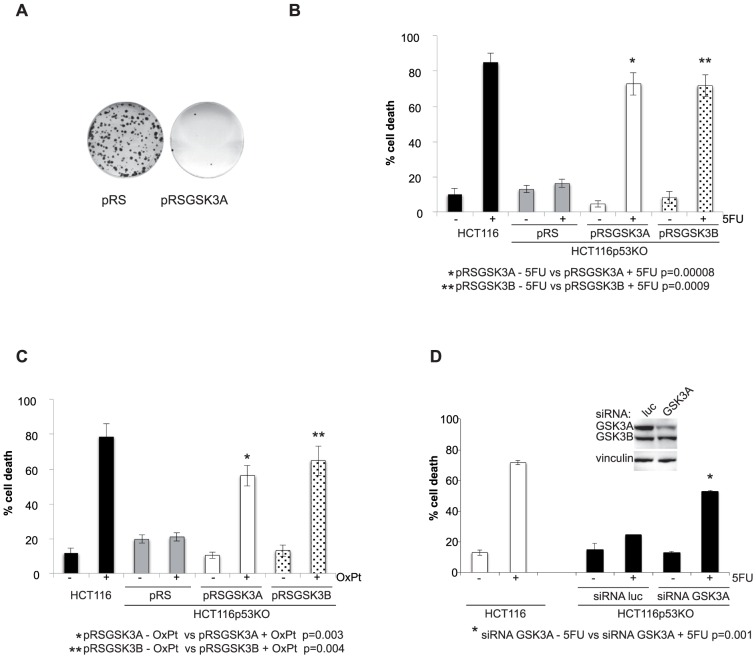
Either stable or transient depletion of GSK3A abolishes drug resistance of p53-null colon carcinoma cell lines. **A**) colony assay of 5FU-treated HCT116p53KO-pRS and -pRSGSK3A cells. Cells were trypsinized and reseeded at low density 12 hours after 200 µM 5FU treatment. Colony formation was assessed 2 weeks after the reseeding. **B**) percentage of cell death of HCT116p53KO-pRS, -pRSGSK3A and -pRSGSK3B cells treated for 72 hrs with 200 µM 5FU. Drug sensitive HCT116 cells were used as a positive control. **C**) percentage of cell death of HCT116p53KO-pRS, -pRSGSK3A and -pRSGSK3B cells treated for 72 hrs with 50 µM Oxaliplatin (OxPt). Drug sensitive HCT116 cells were used as a positive control. **D**) percentage of cell death of HCT116p53KO upon GSK3A transient silencing and treatment with 200 µM 5FU (72 hrs). In the inset: lysates of HCT116p53KO cells transfected with luciferase (luc)- or GSK3A-specific siRNAs were harvested 24 hs after silencing and GSK3A levels checked by western blot.

**Figure 3:**
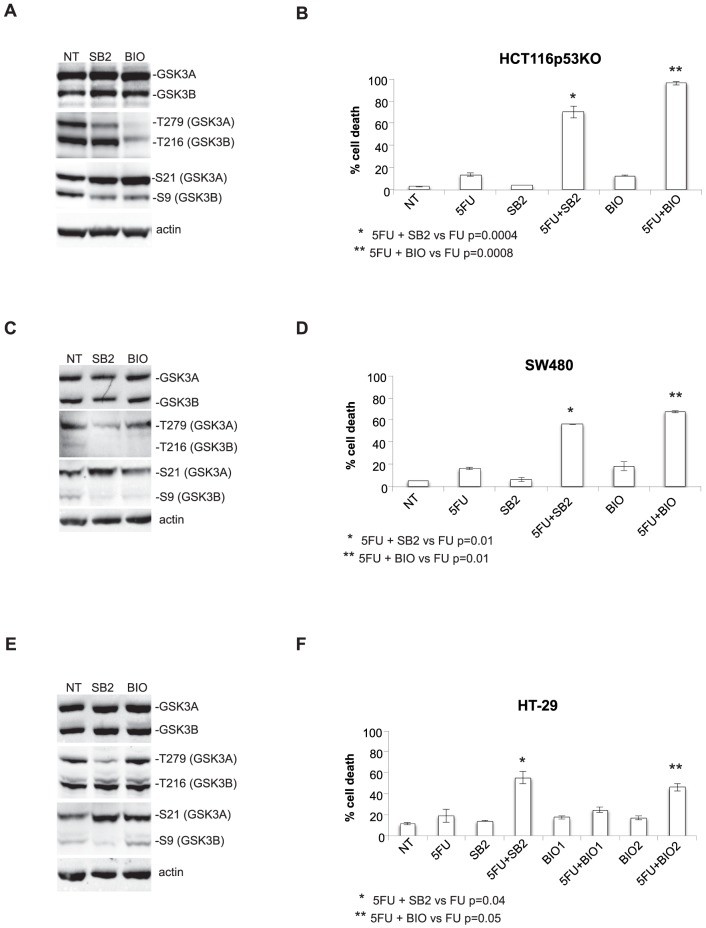
Chemical inhibition abolishes drug resistance of p53-null colon carcinoma cell lines. **A**, **C**, **E** Lysates of HCT116p53KO (**A**) and SW480 (**C**) cells were harvested 24hs after treatment with 1 µM 6-bromoindirubin-3′-oxime (BIO) and 20 µM SB216763 (SB2) GSK3 inhibitors; lysates of HT-29 (**E**) cells were harvested 24 hs after treatment with 2 µM 6-bromoindirubin-3′-oxime (BIO) and 20 µM SB216763 (SB2) GSK3 inhibitors; specificity of the inhibitor for GSK3A was assessed by checking GSK3A activation/inactivation checked by western blot using a mix of pSer21-GSK3A and pSer9-GSK3B antibodies and antibody cross-reacting with both pTyr279-GSK3A and pTyr216-GSK3B. **B**, **D, F**) percentage of cell death of HCT116p53KO(**B**), SW480 (**D**) and HT-29 (**F**) treated with 200 µM 5FU in presence and in absence of the indicated inhibitors (72 hrs).

To test the role of GSK3A in colon carcinoma cells we first established a stable cell line depleted of the protein by transducing drug-resistant HCT116p53KO cells with retroviruses expressing shRNAs to GSK3A (Fig. 1A). We observed that GSK3A stable silencing in HCT116p53KO did not alter cell proliferation: in fact, when comparing shGSK3A and empty vector-transduced HCT116p53KO we did not find significant differences neither in the growth curve (Fig. 1B) nor in cell cycle distribution (Fig. 1C) and β-catenin activation (Fig. 1D). Next, we assessed the role of GSK3A in the response to chemotherapy and found that its depletion in drug-resistant HCT116p53KO cells abolished colony formation after treatment with 200 µM 5-Fluorouracil (5FU) (Fig. 2A). Accordingly, we observed that, in absence of GSK3A expression, HCT116p53KO cells were resensitized to drug-induced cytotoxicity and showed a cell death response similar to that of parental drug-sensitive HCT116 cells (Fig. 2B); same results were obtained in absence of GSK3B expression. Moreover, stable silencing of GSK3A expression reverted the resistance also to OxPt treatment, another DNA-damaging drug commonly used in colon carcinoma therapy. Notably, GSK3A suppression re-sensitized HCT116p53KO cells to the cytotoxic effect of DNA-damaging drugs to the same extent as GSK3B depletion (Fig. 2C). To fully validate the involvement of GSK3A in drug resistance we inhibited its function by 2 more different means [17] i.e., transient silencing and chemical inhibition. As shown in Fig. 2D transient GSK3A protein depletion by use of siRNA restored cell death in response to 5FU. In order to chemically inhibit GSK3A, but not GSK3B, enzymatic activity we first screened a number of commercially available inhibitors (Fig. S1); in fact, being the ATP-binding pockets of GSK3A and GSK3B very similar, most inhibitors block the activity of both isoforms [18]. GSK3 activity is usually kept off by an inhibitory phosphorylation on Ser (S21 for GSK3A and S9 for GSK3B) that has to be removed to allow phosphorylation on Tyr (T279 for GSK3A and T216 for GSK3B), and therefore enzymatic activation, to occur [5]. Using as a readout the phosphorylation pattern of the two isoforms we found that, in our model system, 20 µM SB216763 was able to strongly reduce T279 (even though not completely) and increase S21 phoshorylation without affecting neither T216 nor S9 phoshorylation indicating that at this concentration it specifically inhibits only the GSK3A isoform. At variance, very low concentrations of other inhibitors - such as BIO and TWS - despite increasing the levels of phospho-S21, but not phospho-S9, abolished both T279 and T216 phosphorylation, indicating that these inhibitors affect the activity of both isoforms, perhaps to a different extent. Interestingly, concentrations of BIO able to completely suppress both T279 and T216 phosphorylation (2 µM), were also cytotoxic (Fig. S2) indicating that blocking completely GSK3 activity is not compatible with survival of cancer cells. To confirm that GSK3A inhibition re-sensitize drug-resistant colon carcinoma cells to chemotherapy, we then treated three cell lines characterized by different genetic background, HCT116p53KO, SW480 and HT-29 drug-resistant cells, with 5FU in the presence of 20 µM SB216763 and 1 µM BIO (Fig. 3). Both inhibitors elicited significant cell death in response to DNA-damaging drugs and, consistently with an effect also on GSK3B, higher percentage of cell death was observed in BIO- vs SB216763-treated cells (Fig.3 A–D), with the exception of HT-29 cell line. These cells in fact appeared to be intrinsically more resistant to the effect of BIO (Fig.3 E, F) and the concentration had to be increased to 2 µM to obtain percentages of cell death comparable to those observed for HCT116p53KO and SW480 cells.

On the whole, similarly to what observed upon GSK3B protein blockade, also silencing/inhibiting GSK3A does not affect cell proliferation and only modified the response to DNA-damaging chemotherapy.

### GSK3A inhibition affects the response to DNA damage and elicits RIP1-independent necroptosis in response to 5FU

**Figure 4:**
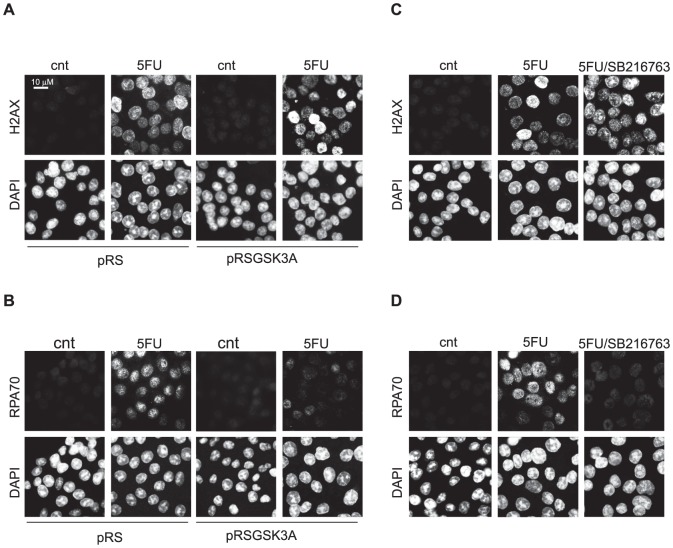
GSK3A inhibition abolishes drug resistance of p53-null colon carcinoma cells by affecting the response to DNA damage. HCT116p53KO cells stably infected with empty (pRS) and GSK3A shRNA-encoding vector (pRSGSK3A) untreated (cnt) or treated for 18 hrs with 200 µM 5FU (5FU) and stained with anti-γH2AX antibody (**A**) or anti-RPA70 antibody (**B**) and counterstained with DAPI. HCT116p53KO untreated (cnt), treated for 18 hrs with 200 µM 5FU (5FU) or with 200 µM 5FU+10 µM SB216763 and stained with anti-γH2AX antibody (**C**) or anti-RPA70 antibody (**D**) and counterstained with DAPI.

Next we investigate whether, similarly to GSK3B, also GSK3A inhibition influences DNA damage response/repair systems. To this end we analysed γH2AX foci formation, as a marker of the DNA damage response [19], and RPA70 foci formation, as a marker of DNA repair [20], in HCT116p53KO cells treated with 5FU. As in the case of GSK3B, also blocking GSK3A, either by silencing (Fig. 4A, B) or by use of a specific inhibitor (Fig. 4C, D) did not affect DNA damage sensing (Fig. 4A, C) as indicated by the formation of γH2AX-positive foci. Similarly to what reported for GSK3B, also GSK3A silencing/inhibition affected the DNA repair response by impairing RPA70 foci formation (Fig. 4B, D).

**Figure 5:**
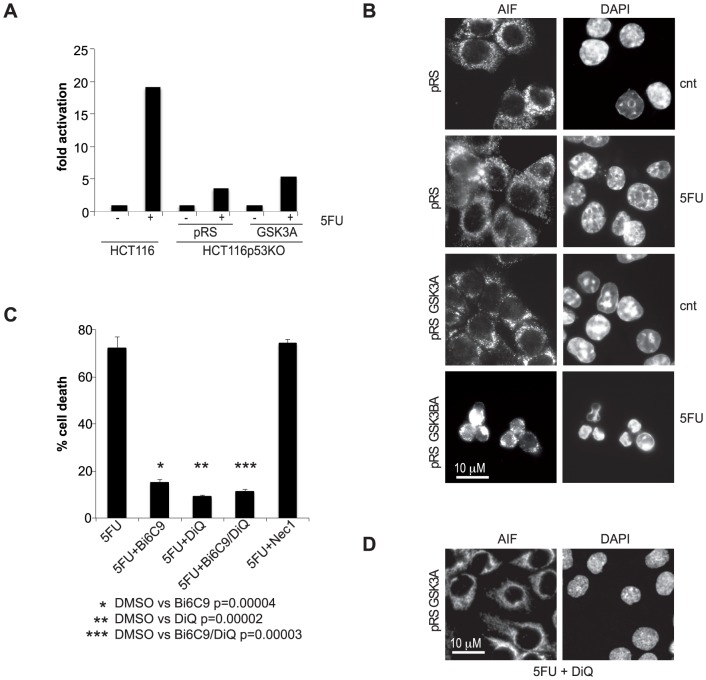
p53-null, GSK3B-silenced colon carcinoma cells treated with 5FU die by RIP1-independent necroptosis. **A**) caspase-3/7 activation in HCT116p53KO-pRS and -pRSGSK3A cells treated with 200 µM 5FU for 72 hrs. HCT116 were used as control. Values indicate the fold increase of enzymatic activity of treated cells relative to the untreated cells arbitrarily set as 1. A representative experiment is shown. **B**) HCT116p53KO-pRS and -pRSGSK3A treated for 30 hrs with 200 µM 5FU and stained with anti-AIF antibody as well as DAPI. **C**) percentage of cell death of HCT116p53KO-pRSGSK3A after 72 hs treatment with 200 µM 5FU in presence of Bid inhibitor (20 µM Bi6C9), PARP1 inhibitor (100 µM DiQ), Bi6C9+DiQ or Necrostatin-1 (20 µM Nec1). **D**) HCT116p53KO-pRSGSK3A treated for 30 hrs with 200 µM 5FU in presence of 100 µM DiQ and stained with anti-AIF antibody as well as DAPI.

Finally, we investigated which kind of cell death is induced in response to DNA-damaging drugs when GSK3A function is blocked in p53-null cells: as in the case of GSK3B inhibition, upon 5FU treatment, GSK3A-depleted HCT116p53KO cells underwent caspase-independent (Fig. 5A), PARP1- and tBid-dependent cell death, which was unaffected by RIP1 inhibition (Fig. 5C) and was characterized by PARP-dependent nuclear re-localization of AIF (Fig. 5B, D).

Altogether our data demonstrate that GSK3A isoform, like GSK3B, contributes to cell survival upon treatment with DNA-damaging drugs by suppressing a necroptotic response.

## Discussion

Few investigations have addressed the role of GSK3A in cancer cells so far. A redundant role with GSK3B has been described in pancreatic cancer cells where both isoforms are involved in NFkB-dependent pro-survival effect [9]. At variance, GSK3A, but not GSK3B, has been identified as a therapeutic target in melanoma [15] suggesting that, similarly to what has been reported for normal cells, the two isoforms can play both distinct or redundant roles depending on the cancer cell type. Our data demonstrate for the first time that GSK3A is redundant with GSK3B in regulating drug-resistance and chemotherapy-induced necroptosis of p53-null colon cancer cells. In addition, they indicate that inhibition of one isoform is sufficient to bypass drug resistance of p53-null colon cancer cells since both GSK3 isoforms negatively regulate the RIP1-independent necroptotic response elicited by chemotherapy.

From our data GSK3 activity appear to negatively regulate PARP1 in response to 5FU (Fig. 3C and D and ref. 16). Notably, PARP1 is involved in three pathways of DNA repair that are differently affected by p53 absence [21] and directly or indirectly activated by 5FU treatment [22]: base excision repair (BER), non-homologous end joining (NHEJ) and homologous recombination (HR). In fact, in absence of wild type p53, activation of BER, the main repair pathway activated by 5FU, is suppressed whereas NHEJ and HR are active leading to aberrant double strand break repair. Accordingly, it has been reported that after severe genotoxic damage, p53 mutant cells can recover from a G2 arrest and resume proliferation upon DNA re-replication during which aberrant DNA repair occurs [23]. Consistent with the data from the literature, in our model system, RPA70 foci -markers of repair activity - are formed in p53-null drug-resistant cells surviving 5FU treatment but they are strongly impaired in absence of GSK3 activity (Fig. 4), when cells are re-sensitized to the cytotoxic effect of chemotherapy. It is likely that following DNA damage and in absence of GSK3 activity PARP activation (Fig. 5C and D) occurs which is not accompanied by DNA repair (Fig. 4B and D), therefore leading to the triggering of cell death mechanisms.

The finding that both isoforms play redundant roles in the response to chemotherapy is particularly relevant both at clinical and therapeutic level. Due to the high similarity in in the ATP-binding pockets of GSK3A and GSK3B, synthesis of inhibitors able to differentiate between the two isoforms is very difficult [18]. Moreover, when targeting GSK3 it has be kept in mind that a complete inhibition of whole GSK3 activity might be undesirable, based on the observation that GSK3A/B double-knockout cells displayed hyperactivated Wnt/β-catenin signaling [7] which may be oncogenic. On the other hand, it also been demonstrated that only in cells lacking three or all four of the alleles a gene-dosage effect was observed, suggesting that there may be a therapeutic window and dose for GSK3 inhibitors in treating diseases without elevating β-catenin levels and thus a risk of oncogenic events [7]. Due to the redundancy played by GSK3A and GSK3B in the response to DNA-damaging drugs it is reasonable to predict that, when used together with anticancer drugs, GSK3 inhibitors would be particularly effective in abolishing drug resistance even at low doses since inhibition of only one isoform, or rather partial inhibition of overall cellular GSK3 activity, is enough to re-sensitize drug-resistant cells. Our data using BIO in combination with 5FU (Fig.3 B, D) strongly support this prediction: in fact, when used together with 5FU, concentrations as low as 1 µM BIO (inhibiting GSK3A and partially GSK3B) induce an higher percentage of cell death cells than 20 µM SB216763 (inhibiting only GSK3A). Moreover, in our model system a low concentration of SB216763 is able to specifically inhibit GSK3A, while several reports demonstrate that in different cell lines and/or models higher concentrations of the same inhibitor blocks also GSK3B function [24]–[27].

In conclusion, we propose that GSK3 inhibition in combination with DNA damaging drugs would be an appealing strategy to induce necroptosis in colon tumors resistant to chemotherapy because of the loss of pivotal apoptosis regulators such as p53.

## Materials and Methods

### Drugs and reagents

5FU (Teva) and Oxaliplatin (Sanofi-Aventis) were from San Gerardo Hospital, Monza. SB216763, SB415286 and necrostatin-1 were from Sigma-Aldrich. 6-bromoindirubin-3′-oxime, TWS119 and Tideglusib were from Selleck Chemicals

### Cell lines and cell culture

Isogenic p53 wild type and p53 knockout HCT116 colon carcinoma cell lines, a kind gift of Dr. Bert Vogelstein (Johns Hopkins University, Baltimore, MD), HT-29 and SW480 were from ATCC. All cell lines were maintained in McCoy medium (Invitrogen) supplemented with 10% fetal bovine serum (Invitrogen) and 1% penicillin-streptomycin at 37°C in 5% CO2. Cell lines stably interfered for GSK3A were obtained by retroviral infection and selection with the appropriate antibiotic as previously described [25]. GSK3A siRNA were purchased from Qiagen (#S100288554), Luciferase siRNA from Eurofins MWG Operon, (#GL2).

### Cell viability

Cells were seeded overnight at 70% confluency and the next morning treated or not with the indicated drugs and inhibitors. 72 hrs later dead cells were counted - triplicate wells in each experiment - after Trypan blue staining. In experiments using inhibitors, after overnight seeding cells were pre-incubated for 2 hrs before 5FU addition. Graphs shown throughout the paper represent the average of three to five independent experiments. Average ±SEMs is plotted in the graphs.

### Colony assay

3×10^5^ cells/well were seeded in 6-well plate, let adhere overnight and treated with 200 µM 5FU for 12 hs. Cells were then trypsinized, counted, and reseeded at a low density (1000 cells/well in 6-well plate) in triplicate; medium was replaced every 3 days, and after 2 weeks colonies were fixed and stained in 1% crystal violet, 35% ethanol.

### Caspase assay

4×10^4^ cells/well were seeded in triplicate in 96-well plate, let adhere overnight and treated with 200 µM 5FU for 72 hrs before evaluating active caspase-3/7 by the Caspase-Glo3/7 Assay System (Promega) according to the manufacturer's instructions.

### Cell proliferation

1×10^4^ cells/well were seeded in triplicate in 96-well plate and starting the following day (day 0) proliferation was evaluated each 24 hrs by CellTiter 96 AQueous Non-Radioactive Cell Proliferation Assay (Promega) according to the manufacturer's instructions.

### Flow Cytometric Analysis

Exponentially growing cells were trypsinized, washed twice with cold PBS, fixed in ice-cold 96% ethanol, washed twice with cold PBS and incubated overnight at 4°C with propidium iodide (10 µg/mL) and RNase A (12.5 µg/mL) in PBS. Fluorescence intensity of 1×10^4^ cells/sample was determined with a FACSCalibur instrument and data analyzed using Modfit Cell Cycle Analysis (Becton Dickinson) as previously described [16].

### Reporter assay

0.2 µg TopFlash +0.2 µg pGL4.75 reporters were transfected in 5×10^4^ cells/well seeded in triplicate in a 96-well plate and reporter activity was evaluated 48 hrs later by Dual-Glo Luciferase Assay (Promega) according to the manufacturer's instructions.

### Western blot analysis

Cells were lysed in high-salt lysis buffer (Hepes 50 mM, pH 7.5, NaCl 500 mM, DTT 1 mM, EDTA 1 mM, 0.1% NP40) supplemented with 1% protease inhibitor cocktail (PIC, Sigma-Aldrich) and Western blots performed as described [25] using the following antibodies: anti-GSK3A/Bsc-7921) and anti-pTyr279/216-GSK3A/Bsc-11758) from Santa Cruz Biotechnology, anti-pSer9-GSK3B (clone D85E12) and anti-pSer21-GSK3A, (clone 36E9) from Cell Signaling, vinculin (SAB4200080) from Sigma-Aldrich.

### Immunofluorescence

Cells were fixed with 4% paraformaldehyde in phosphate-buffered saline. Permeabilization and staining with anti-AIF (sc-13116, SantaCruz Biotechnology), anti-γH2AX (Ab 22551, Abcam), anti-RPA70 (clone 2H10, Sigma-Aldrich) was performed as described [28]. Cells were counterstained with DAPI before microscopic examination using 60× magnification and a Nikon Eclipse 80i microscope. Images were acquired using Genikon (Nikon) software and processed with Adobe Photoshop.

### Statistical analysis

T test was applied to evaluate statistically significant differences between series of samples subjected to different experimental treatments, p≤0.05 was considered significant.

## Supporting Information

Figure S1 **Isoform specificity of different chemical inhibitors of GSK3.** Lysates of HCT116p53KO cells were harvested 24 hs after treatment with different GSK3 inhibitors and GSK3A/B activation/inactivation checked by western blot: a mix of pSer21-GSK3A and pSer9-GSK3B antibodies and antibody cross-reacting with both pTyr279-GSK3A and pTyr216-GSK3B were used to assess the specificity of the inhibitor for GSK3A. BIO: 6-bromoindirubin-3′-oxime, TWS: TWS119, SB2: SB216763, SB4: SB415286.
(EPS)

Figure S2 **Inhibition of both GSK3 isoforms is toxic.** **A**) Lysates of HCT116p53KO and SW480 cells were harvested 24 hs after treatment with 2 µM BIO. Inhibition of GSK3A/B was assessed by western blot using a mix of pSer21-GSK3A and pSer9-GSK3B antibodies and an antibody cross-reacting with both pTyr279-GSK3A and pTyr216-GSK3B. **B**) percentage of cell death of HCT116p53KO and SW480 cells treated in presence and in absence of 2 µM BIO (72 hrs).
(EPS)
